# Safety and Efficacy Assessment of Cannabis Plant Compared to Atorvastatin for Lipid Lowering in Diabetic and Obese Wistar Male Rats

**DOI:** 10.3390/life16081383

**Published:** 2026-08-21

**Authors:** Minela Aida Mărănducă, Andreea Clim, Mariana Floria, Daniela Maria Tănase, Bogdan Tamba, Andrei Szilagyi, Leontina-Elena Filipiuc, Maria Raluca Gogu, Cristian Tudor Cozma, Dragomir Nicolae Șerban, Ionela-Lăcrămioara Șerban

**Affiliations:** 1 Department of Morpho-Functional Sciences II, Discipline of Physiology, Grigore T. Popa University of Medicine and Pharmacy, 700115 Iași, Romania; minela.maranduca@umfiasi.ro (M.A.M.); andreea.clim@umfiasi.ro (A.C.); tudor-cristian.cozma@umfiasi.ro (C.T.C.); dragomir.serban@umfiasi.ro (D.N.Ș.); ionela.serban@umfiasi.ro (I.-L.Ș.); 2 Internal Medicine Clinic, “St Spiridon” County Clinical Emergency Hospital, 700111 Iași, Romania; daniela.tanase@umfiasi.ro; 3 “Dr. Iacob Czihac” Military Emergency Hospital, 700483 Iași, Romania; 4 Advanced Centre for Research-Development in Experimental Medicine, Grigore T. Popa University of Medicine and Pharmacy, 700115 Iași, Romania; bogdan.tamba@umfiasi.ro (B.T.); andrei.szilagyi@umfiasi.ro (A.S.); leontina.filipiuc@umfiasi.ro (L.-E.F.); raluca.gogu@umfiasi.ro (M.R.G.); 5 Department of Pharmacology and Pharmacotherapy, Semmelweis University, 1085 Budapest, Hungary; 6 Cardiology Clinic, “St Spiridon” County Clinical Emergency Hospital, 700111 Iași, Romania

**Keywords:** statins, diabesity, hepatotoxicity, cannabis, tetrahydrocannabinol, cannabidiol, Wistar rat

## Abstract

**Introduction.** Statins’ established cardiovascular benefits are often undermined by hepatic adverse effects in obese diabetic patients with suspected non-alcoholic fatty liver disease. We evaluated whether cannabis plant treatment is a safe and effective alternative to atorvastatin in a metabolically challenged rodent model. **Methods.** Fourteen obese and diabetic Wistar rats received either atorvastatin for 30 days or cannabis plant extract for 30 days. Main outcome: lipid profile; secondary outcomes: renal function, glycemia and endothelial health assessed through atherogenic indices. Safety was assessed using plasma liver enzyme concentrations. **Results.** Baseline biochemical profiles were similar between groups. Atorvastatin increased HDL-Col more than cannabis plant (MD: 44.3; IQR [33, 57.5] mg/dL, Hedges’ *g* 2.713 vs. MD: 14.3; IQR [14, 17] mg/dL, Hedges’ *g* 5.049), and cannabis plant did not change LDL-Col. Atherogenic index of plasma and Castelli Risk Index 1 improved with both interventions, more so with atorvastatin. Liver enzymes increased with atorvastatin (ALAT, MD: 15.9; IQR [12.5, 19.5] U/L; ASAT, MD: 33; IQR [26.5, 38] U/L) but remained virtually unchanged with cannabis. Serum creatinine increased with cannabis plant, with moderate effect size (Hedges’ *g* 0.798), but negligibly with statins (Hedges’ *g* 0.122). **Conclusions.** Cannabis plant extract modestly improved the lipid profile and atherogenic indices, with differences often not exceeding the change in control groups. Despite apparent liver safety, the undesired renal and neurological consequences limit applicability in humans. Further studies should prioritize addiction development and renal function.

## 1. Introduction

Obesity and type 2 diabetes are closely interrelated metabolic disorders that have reached epidemic proportions and are now among the leading public health challenges worldwide [1]. Their coexistence, often referred to as “diabesity”, impairs quality of life, increases morbidity and mortality, and places substantial financial pressure on healthcare systems [2,3]. The central pathophysiological link between obesity and type 2 diabetes mellitus (T2DM) is driven by low-grade chronic systemic inflammation and insulin resistance, both of which stem from excess adipose tissue and adipose tissue dysfunction. These mechanisms are accompanied by marked disruption of lipid homeostasis, including elevated serum triglycerides and LDL-Col levels, together with reduced HDL-Col levels. Lipid dysregulation also contributes to the development of non-alcoholic fatty liver disease, largely through increased hepatic influx of free fatty acids and de novo lipogenesis secondary to hyperinsulinemia. This process is reflected in altered hepatic enzyme profiles and particularly elevated transaminase levels, and may progress toward fibrosis and severe liver dysfunction [4,5]. Diabetic nephropathy represents another chronic complication of obesity-related T2DM and is characterized by severe structural and functional renal impairment. Chronic hyperglycemia and lipid toxicity promote local inflammation, which in turn contributes to interstitial fibrosis and glomerulosclerosis [6]. Plasma atherogenic indices are currently regarded as reliable predictors of cardiovascular risk in metabolic syndrome because they reflect the balance between atherogenic and antiatherogenic lipid fractions [7].

In this context, atorvastatin is widely used as a first-line treatment for dyslipidemia and for reducing cardiovascular risk associated with metabolic syndrome. By competitively inhibiting HMG-CoA reductase, atorvastatin reduces hepatic cholesterol synthesis and enhances the clearance of atherogenic plasma fractions [8,9]. In addition to its lipid-lowering effects, atorvastatin has also been reported to exert renoprotective properties especially relevant during the earlier stages of chronic kidney disease, without causing harm in advanced stages [10,11,12]. Despite their proven efficacy in reducing atherogenic plasma markers, statins have several limitations, particularly regarding the management of systemic inflammation and non-alcoholic fatty liver disease when diabetes is present [13]. Moreover, the muscular and hepatic adverse effects associated with high-dose statin therapy highlight the need for alternative or complementary therapeutic strategies.

Because of its direct involvement in the regulation of energy balance, lipid metabolism, and carbohydrate metabolism through both central and peripheral mechanisms, modulation of the endocannabinoid system has emerged as an innovative, although still controversial, therapeutic field. This controversy is largely related to the psychoactive effects of cannabinoid compounds [14,15]. Delta-9-tetrahydrocannabinol (THC), the main bioactive compound of *Cannabis sativa*, acts as a partial agonist at CB1R and CB2R cannabinoid receptors. Activation of CB1R alters glucose metabolism at the hepatocyte level through induction of the liver-specific transcription factor cAMP-responsive element-binding protein H [16]. Furthermore, obesogenic conditions switch the effect of CB1R stimulation at the hepatocyte level towards lipogenesis and steatotic liver disease [17]. Downregulation of CB1R has been achieved through chronic THC administration and has been linked to improved lipid profiles, reduced insulin resistance, and decreased hepatic lipogenesis [18,19,20]. Adding to the potential benefit of CB1R downregulation, hyperactivation of the renal endocannabinoid system in obesity and diabetes may amplify urea retention and contribute to progressive increases in creatinine levels through podocyte injury [21,22]. Peripheral CB1R blockade may reverse the effect of obesogenic diet [23]. Nevertheless, the mechanisms underlying these effects remain incompletely understood, and the reported outcomes are often contradictory [24,25].

At present, no studies have directly compared classical lipid-lowering therapy with atorvastatin and THC in terms of their metabolic, renal, and hepatoprotective potential. Still less known are the cardiovascular actions of cannabis-derived compounds, but the literature provides neutral evidence in this regard [26,27]. Aside from their limited-application pain-relieving properties, cannabis derivates’ effects on endothelial health are a less researched area. Therefore, the aim of this study was to evaluate and compare the effects of atorvastatin—a staple of cardiovascular preventive treatment—and cannabis extract upon endothelial function—assessed indirectly through lipid profile and atherogenic indices—in an obese and diabetic male Wistar rat model. Intervention safety was assessed using liver enzymes and fibrosis-focused derived indices. By comparing these interventions, the study may provide insight into the therapeutic potential and limitations of atorvastatin and cannabis in the realm of metabolic syndrome.

## 2. Materials and Methods

### 2.1. Animals

A total of 35 six-month-old male Wistar rats, weighing 350–400 g, were obtained from the Cantacuzino Institute (Bucharest, Romania). The animals were part of a larger research project investigating obesity-related endothelial dysfunction. Throughout the study, they were housed at the Advanced Research and Development Center for Experimental Medicine (CEMEX) of the “Grigore T. Popa” University of Medicine and Pharmacy in Iași, Romania [28,29].

The animals were maintained under controlled environmental conditions, with an ambient temperature of 20 ± 2 °C, relative humidity of 50 ± 5%, and a 12 h light/dark cycle. Rats were housed in pairs in individually ventilated cages and provided ad libitum access to water and their assigned diets. Environmental enrichment was provided throughout the study using nesting material, and animal health and welfare were monitored daily for signs of pain, distress, or illness. Following a two-week acclimatization period, the animals were randomly assigned to experimental groups, after which, the study procedures were initiated.

### 2.2. Ethical Approach

All experimental procedures were conducted in accordance with the European Union Directive 2010/63/EU on the protection of animals used for scientific purposes and Romanian legislation (Law No. 43/2014). The study protocol was reviewed and approved by the Ethics Committee of the “Grigore T. Popa” University of Medicine and Pharmacy of Iasi (Approval No. 277/26 February 2023) and by the Romanian National Sanitary Veterinary and Food Safety Authority (Approval No. 61/27 April 2023).

### 2.3. Experimental Design and Animal Treatment Groups

This experimental model was selected because it reproduces, under controlled conditions, several metabolic alterations characteristic of human obesity-associated T2DM. To investigate the effects of atorvastatin and cannabinoids on the glycemic profile, lipid metabolism, and renal and hepatic function, the 35 rats were randomly assigned to five experimental groups (n = 7 per group). Before the start of the study, all animals underwent a 14-day acclimatization period under standardized laboratory conditions to minimize stress and allow adaptation to the new environment. Group I served as the healthy control group and received a standard laboratory diet throughout the study. Group II consisted of obese animals that received a High-Fat Diet (HFD) supplemented with 2% cholesterol for one month prior to study initiation. Likewise, rats assigned to Groups IV and V were fed the same cholesterol-enriched HFD for one month to induce obesity, after which, type 2 diabetes mellitus was experimentally induced. The experimental diet was prepared by the Cantacuzino Institute (Bucharest, Romania) according to a previously described protocol. T2DM was induced in Groups III-V by a single Streptozotocin injection, as described below. Once diabetes was confirmed in all 21 specimens, 14 rats designated for treatment were randomly assigned to G-IV and G-V; animals in Group IV received atorvastatin at a dose of 20 mg/kg body weight. The treatment was administered orally by gavage once daily for 30 consecutive days. Pre-filled syringes were prepared prior to animal handling, and the person responsible for treatment administration was blinded to animal group membership and treatment option. Animals in Group V received cannabinoids at a dose of 3 mg/kg body weight following the same administration schedule. All animals reached the end of the experiments.

The first biochemical analyses were performed on day 54 after the animals’ arrival at the laboratory using blood samples collected from all animals by retro-orbital puncture of the right orbital venous plexus.

Gross necropsy was performed on day 85 after arrival at the laboratory, corresponding to 31 days after treatment initiation. After a 12 h fasting period, rats were anesthetized with isoflurane (5% for induction and 1.8–2.5% for maintenance) and subsequently euthanized by isoflurane overdose. This euthanasia method was selected in accordance with the published literature addressing the effects of euthanasia techniques on blood-derived parameters [30,31,32,33,34]. Terminal blood samples were then collected by cardiac puncture, obtaining approximately 4 mL from each animal into clot-activator vacutainer tubes for biochemical analyses. A complete gross necropsy was then performed, including systematic examination of the external body and internal cavities, followed by targeted tissue collection for further analyses. Group design is summarized in Table 1.

### 2.4. Induction of T2DM in an Experimental Model of Rats

After 30 days of HFD feeding, T2DM was induced in Groups III, IV, and V by a single intraperitoneal injection of Streptozotocin (STZ; 35 mg/kg body weight). Blood glucose levels were measured on days 7 and 10 after STZ administration (corresponding to 51 and 54 days after specimen arrival in laboratory), and successful diabetes induction was confirmed on day 10 based on glycemic values, as described in a previous study [11]. All 21 specimens survived the procedure and developed T2DM, thus entering the intervention phase of the experiment.

### 2.5. Preparation of Solutions for Interventions

The cannabis preparation used in this study was obtained from Cansativa GmbH (Mörfelden-Walldorf, Germany) and consisted of EU GMP-certified Cannabixir^®^ Me-dium Flos (PZN: 7001905), consisting of dried inflorescences of Cannabis sativa L., containing 15.6% Δ9-tetrahydrocannabinol and less than 1% cannabidiol (CBD). The dried plant material was finely pulverized using an RM 200 laboratory grinder (Retsch GmbH, Haan, Germany) and subsequently passed through a 125 µm sieve (BSS Mesh No. 120) to ensure a homogeneous particle size. To facilitate oral administration, the resulting powder was suspended in 0.1% sodium carboxymethyl cellulose. Fresh suspensions were prepared on a weekly basis, stored at 2–8 °C, and allowed to equilibrate to room temperature immediately before administration.

Cannabis treatment was administered by oral gavage at 3 mg/kg body weight/day for 30 consecutive days, following the experimental protocol. This dose was selected based on a previous study reporting no organ damage at 5 mg/kg body weight/day [35].

Atorvastatin (Sortis^®^, Pfizer; New York, USA, Batch No. NM4027) was crushed into a fine powder using a mortar, then dissolved in distilled water before each administration. The drug was delivered orally by gavage using sterile 16 G × 1.5 feeding needles (Popper and Sons, New York, USA), with a separate feeding device assigned to each animal to prevent cross-contamination.

To minimize potential variability associated with the gavage procedure and handling, rats from the rest of the experimental Groups I to IV received an equivalent volume of distilled water using the same administration technique.

### 2.6. Complete Biochemical Assay Protocol

Cages for each group of animals could be identified by the handling person, and the animals in Groups III to V were objectively larger than the other two groups. Within 30 min of blood collection, vacutainer tubes were centrifuged at 1500× *g* for 15 min at 4 °C. The biochemical panel included renal function markers (urea and creatinine), lipid profile parameters (total cholesterol, HDL cholesterol, LDL-cholesterol, and triglycerides), liver enzymes (ASAT and ALAT), and glycemia. All measurements were performed using the ACCENT-S120 automated analyzer (PZ Cormay, Warsaw, Poland), following the manufacturer’s protocols. Reagents for the standard biochemical assays were obtained from Cormay Diagnostics (PZ Cormay, Warsaw, Poland). We also employed the use of derived indices for atherogenic profile: atherogenic index of plasma (AIP), Castelli Risk Index 1 and 2 (CRI-1,2); for renal function, blood urea nitrogen (BUN) derived by serum creatinine (sCr); and for liver function, de Ritis ratio, and ASAT derived by square root of ALAT. All indices were based on commonly available formulas [7,36].

### 2.7. Statistical Analysis

Biochemical marker distributions were visualized with box plots generated in Python 3.11 using Seaborn version 0.13.2. Parameters were plotted by experimental group at the initial and final time points. Each box represents the 25th–75th percentiles, the central line the median, the X the mean, whiskers 1.5× IQR, and individual points the outliers.

Given the small sample size, we assumed that group values were not normally distributed. Within-group Before–After comparisons were performed using the paired-sample Wilcoxon signed-rank test, with a two-tailed significance threshold of *p* = 0.05. After identifying potential experimental effects, we calculated the After-minus-Before difference for each specimen and summarized these values as the mean difference (MD) with the interquartile range (IQR). Differences between the two intervention groups were compared using the independent-sample Wilcoxon test at a significance level of *p* = 0.05. Values were visualized as percentages of the corresponding baseline measurements. Finally, intervention effect sizes were reported for relative differences (adjusted to baseline) using Hedges’ *g*, with continuity correction where appropriate [37]. Overall intervention effectiveness was interpreted by integrating the MD with Hedges’ *g*, because effect size estimates are influenced by value dispersion, as reflected by the standard deviation (SD).

Sample size estimation was based on the expected reduction in triglycerides following atorvastatin treatment. Reference values were drawn from our previous work and the supporting literature, and a minimum anticipated difference of 25 mg/dL was assumed for the atorvastatin dose and treatment duration used in this study. The cannabis-treated group was assigned the same sample size to allow direct comparison between interventions. Unpublished data from similar obese and diabetic rodent models indicated a relatively uniform distribution of triglyceride values across groups, with a maximum standard deviation of 15 mg/dL. Using 80% statistical power and a 5% type I error rate (alpha), the minimum required sample size was set at seven rats per group. For ethical reasons, each experimental group included seven animals.

## 3. Results

**Figure 1:**
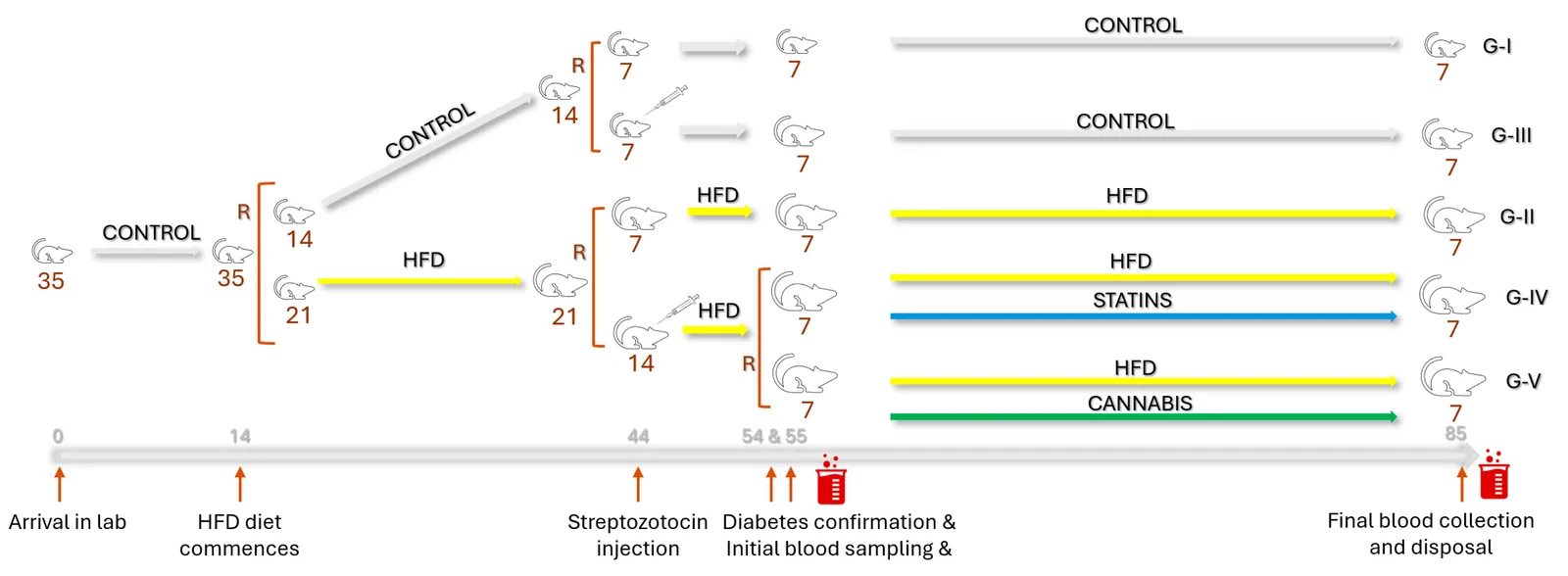
Schematic representation (horizontal axis, days) of randomization processes which took place during our experiment. Control, control diet; HFD, High-Fat Diet; R, randomization occurrence.

All 35 specimens achieved the end point of the study and were disposed of in accordance with the ethical protocol. Numerical data can be found in Supplementary Materials. The schematic representation of the experiment course is found in Figure 1.

### 3.1. Randomization Quality Assessment for Treated Groups

Following all models’ induction and randomization, the resulting Groups IV and V were the only two similar at baseline, both obese and diabetic. To check for any potential bias resulting from randomization of these 14 specimens, we compared the whole biochemical profile and derived indices at the initial point. We did not encounter any clinically or statistically significant differences between Groups IV and V at baseline, following randomization for further treatment.

### 3.2. Before–After Comparison of Biochemical Profile

We performed a within-group comparison for each of the five groups using a Before–After time point difference.

Glycemia-wise, only G-IV showed a statistically significant and clinically relevant decrease after the experiment, a mean difference in MD: −31; IQR [−45; −28] mg/dL. G-V did not achieve a statistically or clinically meaningful difference between the two points in time, MD: 13.86; IQR [−6; 36] mg/dL; neither did the first three groups, G-I to G-III.

LDL-Col values showed a statistically significant reduction at the end of the study for G-III of MD: −17.4; IQR [−25; −10] mg/dL, but this difference does not hold clinical relevance considering the high starting values for this parameter in G-III (mean initial value of 176.6 mg/dL, relative mean difference < −10%). None of the other four groups showed clinically (absolute MD < 11 mg/dL, and relative MD < 5% in the left groups) or statistically significant differences between the two study points for LDL-Col.

HDL-Col showed an increase in mean values across all groups, all statistically significant, but the highest improvement was recorded in G-IV, of MD: 44.3; IQR [33; 57.5] mg/dL. Still, G-V returned a clinically relevant and statistically significant increase in MD: 15.9; IQR [14; 17] mg/dL, a relative MD increase of +29.5%.

Regarding total cholesterol, G-I and G-V did not show a clinically (relative MD for G-I < 3%, relative MD for G-V < 1%) nor statistically significant difference following the study. However, G-II and G-III showed a statistically significant decrease following the experiment, albeit with limited clinical implication: G-II, MD: −22.1 mg/dL, mean starting value 247.3 mg/dL, overall relative MD of −8.9%; G-III, MD: −19.6 mg/dL, mean starting value 292.6 mg/dL, overall relative MD of −6.7%. G-IV was the only one to show an increase at the end of the experiment compared to initial values, and this difference attained statistical and clinical significance, MD: 38.3; IQR [18; 53.5] mg/dL, relative MD of +14%, increase compared to baseline.

Triglyceride-wise, G-II and G-IV returned a statistically significant difference following the experiment, both showing lower average values compared to starting time point. Despite statistical significance, the MD: −7.9; IQR [−10.5; −4] mg/dL from G-II has limited clinical significance considering the average starting value of 199.4 mg/dL, a relative MD of −3.9%, decrease after the experiment. G-IV showed an MD: −38.6; IQR [−46; −20] mg/dL, a −15.2%, decrease with statin treatment. G-V showed a modest, not statistically significant, increase following the study, MD: 10.6; IQR [−11; 36] mg/dL.

Urea values showed a statistically significant and clinically relevant decrease in G-III and G-IV, of MD: −35.1; IQR [−47; −26] mg/dL and MD: −33; IQR [−39.5; −26] mg/dL, respectively. G-V showed a modest decrease in urea values following treatment with cannabis plant, MD: −3.3; IQR [−9; 2] mg/dL, an MD of −7.0% relative to baseline values, without clinical or statistical significance.

Creatinine values did not show a statistically significant difference in any of the five groups. However, the mean difference in MD: 0.26; IQR [0.05; 0.40] mg/dL in G-V is clinically relevant, translating to a relative MD of +29.34% adjusted for baseline values. In G-IV, a mean difference in MD: 0.03 mg/dL voids clinical relevance.

ALAT, although carrying potential bias concerning randomization at experiment initiation, showed a statistically significant and clinically relevant increase in G-IV, an MD: 15.9; IQR [12.5; 18.5] mg/dL. The other groups showed clinically irrelevant differences between the two study points (absolute MD < 3 U/L in all other groups).

**Figure 2:**
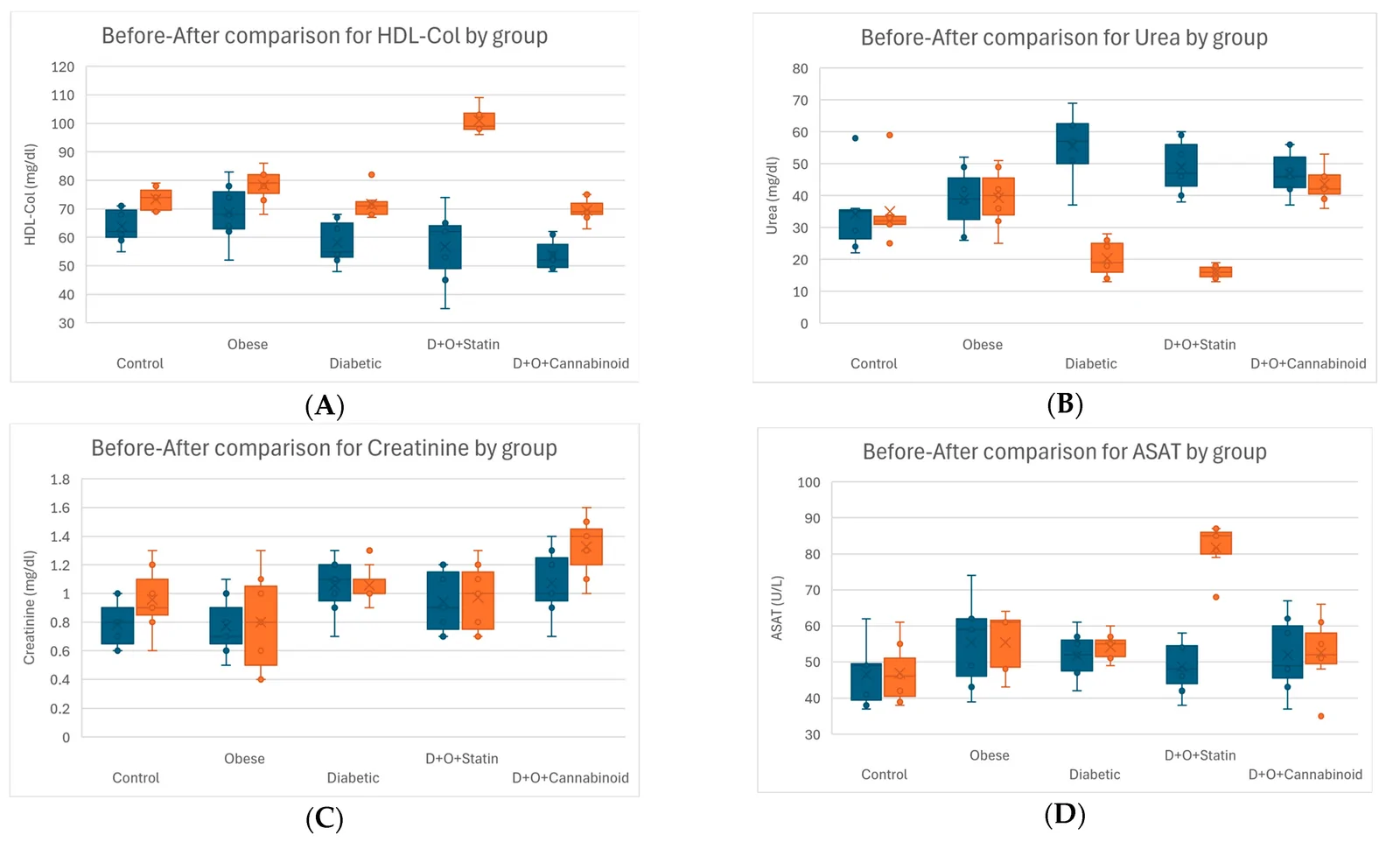
Before–After comparison of basic biochemical profile. Blue boxplots, initial values. Orange boxplots, final values. Plot per each of the five groups is identified below each pair of boxplots.

ASAT, similar to ALAT, showed a statistically significant and clinically relevant increase in G-IV, of MD: 33; IQR [26.5, 38] mg/dL. The rest of the groups did not return clinically relevant differences. The Before–After comparison for HDL-Col, urea, creatinine and ASAT are depicted in Figure 2A–D below, sorted by group.

### 3.3. Intervention Effect Comparison by Biochemical Profile

The effects of statin versus cannabis treatments on a Before–After time frame were first assessed by comparing the after-treatment values, adjusted for baseline (difference per specimen, report as percentage from initial value). At the same time, we compared the effect sizes (which inherently depend on spread around mean) within-sample between the two interventions solely, to account for the heterogeneity of the treatment effectiveness.

#### 3.3.1. LDL-Cholesterol

LDL-Col was the one parameter to show the most similar effects concerning the two interventions (*p* = 0.375). Effect size was small in both cases (in G-IV, Hedges’ *g* = 0.104; in G-V, Hedges’ *g* = 0.262), mainly as a result of high standard deviation values for Before–After paired-sample difference in both groups.

#### 3.3.2. Creatinine

Creatinine values were the only other basic biochemical parameter to lack statistical significance for the comparison of the two interventions (*p* = 0.08), although the increase in plasma creatinine concentration for G-V (MD: 0.26; IQR [0.05; 0.40] mg/dL) versus the slight change for G-IV (MD: 0.03; IQR [−0.15; 0.15] mg/dL) are vastly different from a clinical perspective. The effect size was moderate for G-V (Hedges’ *g* = 0.798), but negligible for statin treatment (Hedges’ *g* = 0.122). The moderate effect size in G-V translates to a relative MD of 29.3%, an increase after treatment.

Of the three untreated groups, the largest relative MD was noted for G-I control, a relative MD of 25.3% increase after the experiment. Comparison of Before–After relative changes between G-I and G-V did not yield statistical significance (*p* = 0.95).

All seven other basic biochemical parameters showed statistically significantly different mean differences between the two intervention arms.

#### 3.3.3. Glycemia

Glycemia values were improved by statin treatment in G-IV, with an effect size of Hedges’ *g* = −1.459, denoting high effectiveness towards lower values. On the other hand, G-V returned a moderate effect size of Hedges’ *g* = 0.403, towards increased glycemia. Comparison of the Before–After relative changes for the two interventions yielded statistical significance (*p* = 0.012).

Of the three untreated groups, the largest relative MD was noted for G-II, obese, a relative MD decrease of −8.5% at the end of the experiment, followed by G-III diabetics, a relative MD decrease of −5.3% decrease at the end of the study. Comparison of Before–After relative changes between G-II, G-III and G-IV did not yield statistical significance (G-II vs. G-IV, *p* = 0.48; G-III vs. G-IV, *p* = 0.95).

#### 3.3.4. HDL-Cholesterol

HDL-Col values reveal an apparently contradicting effect between treatments.

The Before–After paired-sample differences in G-IV show a large MD: 44.3; IQR [33, 57.5] mg/dL with statin treatment, in comparison to the more modest MD: 14.3; IQR [14, 17] mg/dL with cannabinoids. However, dispersion around the mean difference value is responsible for the inverse dimension of the effect. Both interventions returned a high effect size, but in G-V, the Hedges’ *g* = 5.049 far surpassed the Hedges’ *g* = 2.713 in G-IV. The comparison of Before–After relative changes between interventions was statistically significant (*p* < 0.001).

Of the three untreated groups, the largest relative MD was noted for G-III, diabetics, a relative MD increase of 24.3% after the experiment. Comparison of Before–After relative changes between G-III and G-IV yielded statistical significance (*p* < 0.01). Comparison of Before–After relative changes between G-III and G-V did not yield statistical significance (*p* = 0.17). Comparison of Before–After relative changes between G-I, G-II and G-V yielded statistical significance (*p* < 0.01).

#### 3.3.5. Total Cholesterol

COL-Total values were increased with statin intervention, a Before–After paired sample difference of MD: 38.3; IQR [18, 53.5] mg/dL, and a large effect size of Hedges’ *g* = 1.310. In G-V, COL-Total values were virtually unchanged; the effect size of Hedges’ *g* = −0.008. The comparison of Before–After relative changes between interventions was statistically significant (*p* = 0.03).

Of the three untreated groups, the largest relative MD was noted for G-II, obese, a relative MD of −9.0% decrease after the experiment. G-IV was the sole group in which COL-Total achieved a significant increase.

#### 3.3.6. Triglycerides

Triglyceride values were decreased with statin intervention, a Before–After paired-sample difference in G-IV of MD: −38.6; IQR [−46, −20] mg/dL. In comparison, the same difference in G-V was MD: 10.6; IQR [−6.5, 26] mg/dL. In G-IV, the effect size was large, Hedges’ *g* = −1.173, in comparison to G-V Hedges’ *g* = 0.435. The comparison of Before–After relative changes between interventions was statistically significant (*p* < 0.01).

Of the three untreated groups, G-I returned a relative MD increase of 0.9%, G-II a relative MD decrease of −4.1%, and G-III a relative MD decrease of −4.2% after the experiment. Comparison of Before–After relative changes between G-II, G-III and G-IV did not yield statistical significance (G-II vs. G-IV, *p* = 0.11; G-III vs. G-IV, *p* = 0.75). Comparison of Before–After relative changes between G-I and G-V did not yield statistical significance (*p* = 0.41).

#### 3.3.7. Plasma Urea

Urea values were decreased by both interventions, but the magnitude was significantly higher in G-IV (MD: −33; IQR [−39.5, −26] mg/dL) compared to G-V (MD: −3.29; IQR [−7.5, 1] mg/dL). The effect size with statins was large, Hedges’ *g* = −3.710, compared to a more modest Hedges’ *g* = −0.595 with cannabis plant extract. The comparison of Before–After relative changes between interventions was statistically significant (*p* < 0.001).

Of the three untreated groups, the largest relative MD was noted for G-III, diabetics, a relative MD decrease of −61.9% after the experiment. Comparison of Before–After relative changes between G-III and G-IV did not yield statistical significance (*p* = 0.48). Comparison of Before–After relative changes between G-III and G-V did not yield statistical significance (*p* = 0.17). Comparison of Before–After relative changes between G-I and G-V did not yield statistical significance (*p* = 0.48).

#### 3.3.8. ALAT Circulating Concentrations

ALAT plasma values significantly increased after statin treatment, MD: 15.9; IQR [12.5, 19.5] U/L. In contrast, cannabis plant treatment did not alter ALAT values after intervention to a clinically relevant extent (MD: −2.3; IQR [−3, −2] mg/dL). However, value dispersion for Before–After paired-sample analysis was far narrower in G-V, leading to a large effect size of Hedges’ *g* = −2.858, contrasting to the relatively smaller—but absolutely large—effect size with statins in G-IV of Hedges’ *g* = 1.191.

Of the three untreated groups, the largest relative MD was noted for G-III, a relative MD of −2.0% decrease after the experiment, followed by G-II with a relative MD of −1.0% decrease. Comparison of Before–After relative changes between G-I and G-IV yielded statistical significance (*p* < 0.001). Comparison of Before–After relative changes between G-III, G-II, G-I and G-V did not yield statistical significance (*p* > 0.05).

#### 3.3.9. ASAT Circulating Concentrations

ASAT plasma values significantly increased after statin treatment, MD: 33; IQR [26.5, 38] U/L. In contrast, cannabis plant extract did not alter ASAT values after intervention to a clinically relevant extent (MD: 0.6; IQR [−1.5, 5] mg/dL). Effect size with statins was large, Hedges’ *g* = 3.177, in comparison to a negligible effect size with cannabinoids in G-V of Hedges’ *g* = 0.174.

Of the three untreated groups, the largest relative MD was noted for G-III, a relative MD increase of +5.5% after the experiment. Comparison of Before–After relative changes between G-III and G-IV yielded statistical significance (*p* < 0.001). Comparison of Before–After relative changes between G-I and G-V did not yield statistical significance (*p* = 0.70).

**Figure 3:**
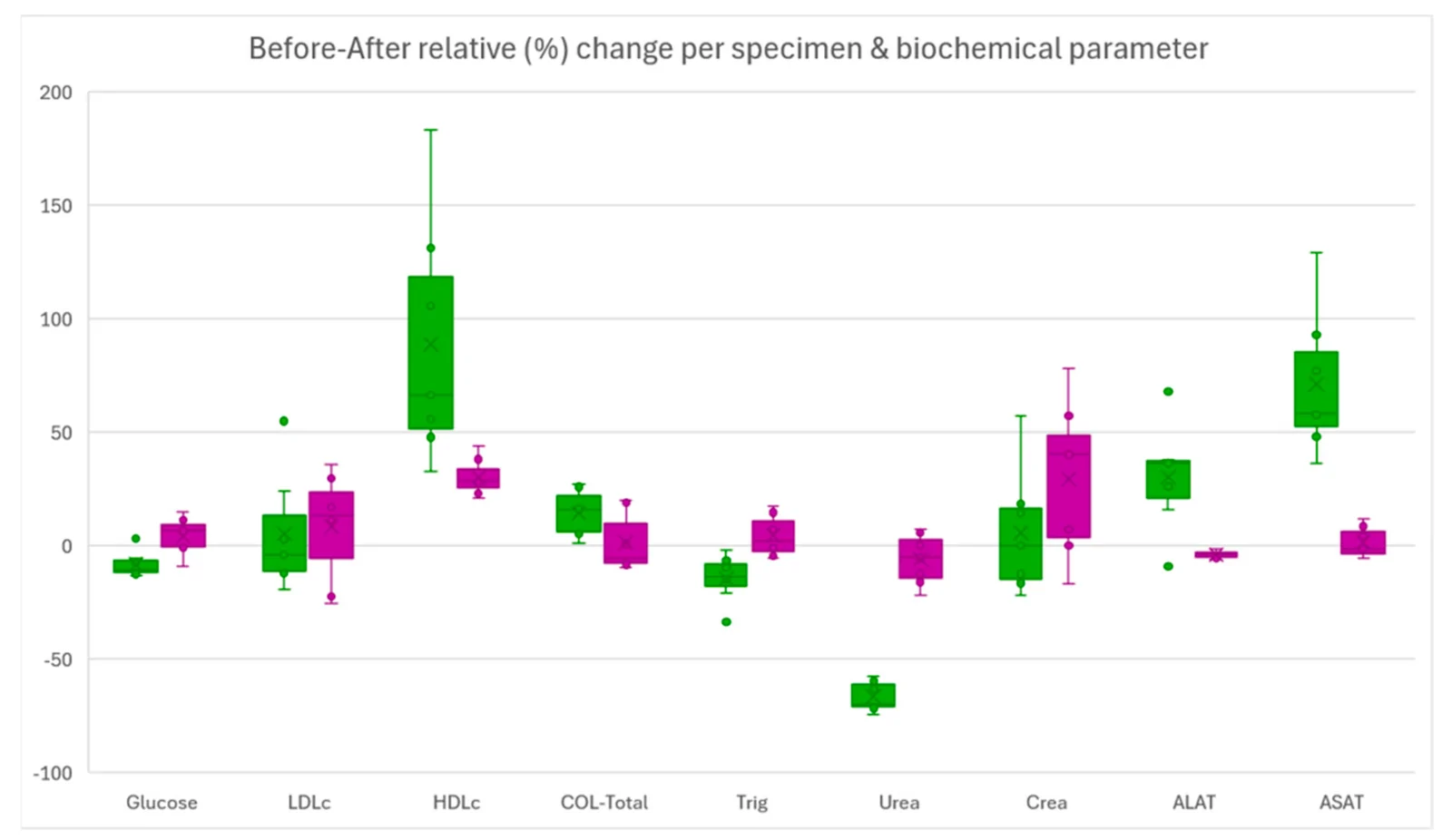
Before–After relative change per specimen, plot by group for each biochemical parameter. Green boxplots, G-IV treated with atorvastatin. Purple boxplots, G-V treated with cannabis plant.

The Before–After paired-sample differences can be visualized in Figure 3 below. We performed per-specimen Before–After difference, divided by the specimen’s initial value for that parameter; then, we plotted the obtained values (by percentage) for both interventions.

### 3.4. Before–After Comparison for Derived Indices of Endothelial and Renal Function

Concerning endothelial health status, we included the AIP, CRI-1 and CRI-2 indices.

#### 3.4.1. Atherogenic Index of Plasma

AIP showed statistically significant differences in all groups except for control, with lower values at the end of the experiment (*p* < 0.05). Effect size was large in both treatment groups, as shown by a Hedges’ *g* = −2.012 in G-IV and a Hedges’ *g* = −1.852 in G-V. The mean of differences with statins (MD: −0.318; IQR [−0.491; −0.149]) was larger than with cannabis plant (MD: −0.096; IQR [−0.115; −0.080]). The comparison of Before–After absolute changes between interventions was statistically significant (*p* = 0.004).

Of the three untreated groups, the largest absolute mean difference was noted for the diabetic G-III, of MD: −0.119; IQR [−0.162; −0.011], followed by the obese G-II, of MD: −0.078; IQR [−0.134; −0.051]. The comparison of Before–After absolute changes between G-III and G-IV was statistically significant (*p* = 0.047). The comparison of Before–After absolute changes between any of the untreated groups G-I to G-III and G-V was not statistically significant (*p* > 0.05).

Of the three untreated groups, the largest relative MD was noted for G-I, a relative MD of −22.7% decrease after the experiment, followed by G-III with a relative MD of −18.6% decrease. Comparison of Before–After relative changes between G-I and G-IV yielded statistical significance (*p* = 0.042). Comparison of Before–After relative changes between any of the untreated groups G-I to G-III and G-V did not yield statistical significance (*p* > 0.05).

#### 3.4.2. Castelli Risk Index 1

From a Before–After within-group perspective, CRI-1 showed statistically significant differences in all groups, including the control, with statistically significant lower values at the end of the experiment (*p* < 0.05). Based on Before–After absolute differences, comparison of effect size showed a not statistically significant difference between the effect of statin versus cannabis plant intervention (*p* = 0.204). Effect size was large in both groups, as shown by a Hedges’ *g* = −1.309 in G-IV and a Hedges’ *g* = −2.028 in G-V. The mean of differences with statins (MD: −1.980; IQR [−3.886; −0.845]) was higher than with cannabis plant extract (MD: −1.227; IQR [−1.862; −0.858]).

Of the three untreated groups, the largest absolute mean difference was noted for the diabetic G-III, of MD: −0.130; IQR [−0.178; −0.841], followed by the obese G-II, of MD: −0.812; IQR [−1.637; −0.396]. The comparison of Before–After absolute changes between G-III and G-IV was statistically significant (*p* = 0.047), unlike the comparison between G-II and G-IV (*p* = 0.110). The comparison of Before–After absolute changes between any of the untreated groups G-I to G-III and G-V was not statistically significant (*p* > 0.05).

Of the three untreated groups, the largest relative MD was noted for G-III, a relative MD of −24.4% decrease after the experiment, followed by G-II with a relative MD of −20.2% decrease. Comparison of Before–After relative changes between G-III and G-IV did not yield statistical significance (*p* = 0.142); neither did those between G-II and G-IV (*p* = 0.064). Comparison of Before–After relative changes between any of the untreated groups G-I to G-III and G-V did not yield statistical significance (*p* > 0.05).

#### 3.4.3. Castelli Risk Index 2

CRI-2 showed a heterogeneous direction of effect at the end of the experiment. With statins, CRI-2 returned lower values, but without statistical significance on a Before–After absolute values comparison (*p* = 0.205). With cannabis plant extract, CRI-2 returned higher values, but without statistical significance on a Before–After absolute values comparison (*p* = 0.554). Based on Before–After absolute differences, effect size was modest in both cases, with a Hedges’ *g* = −0.569 for statins and a Hedges’ *g* = 0.296 for cannabis plant extract. The comparison of Before–After absolute changes between interventions was statistically significant (*p* = 0.04).

Before–After difference was situated on both sides of the null value with both interventions, yielding further comparisons pointless.

#### 3.4.4. Blood Urea Nitrogen to Creatinine Ratio

BUN/creatinine ratio showed a statistically significant difference after the experiment in the groups G-III to G-V, with lower values at the end of the experiment (*p* < 0.05). With statins, the BUN/creatinine effect size was large, Hedges’ *g* = −2.126, and a mean of differences MD: −17.6; IQR [−25.2; −12.8] (adymensional). With cannabis plant extract, the BUN/creatinine effect size was still large, Hedges’ *g* = −1.117, and a mean of differences MD: −5.6; IQR [−9.2; −0.2] (adymensional). The comparison of Before–After absolute changes between interventions was statistically significant (*p* = 0.002).

Of the three untreated groups, the largest absolute mean difference was noted for the diabetic G-III, of MD: −15.8; IQR [−18.0; −14.4], followed far by the control G-I, of MD: −3.3; IQR [−11.2; 1.1]. The comparison of Before–After absolute changes between G-III and G-IV was not statistically significant (*p* = 0.949). The comparison of Before–After absolute changes between G-I and G-V was not statistically significant (*p* = 0.406).

Of the three untreated groups, the largest relative MD was noted for G-III, a relative MD of −62.5% decrease after the experiment, followed by G-I with a relative MD of −10.5% decrease. Comparison of Before–After relative changes between G-III and G-IV did not yield statistical significance (*p* = 0.848). Comparison of Before–After relative changes between G-I and G-V did not yield statistical significance (*p* = 0.277).

**Figure 4:**
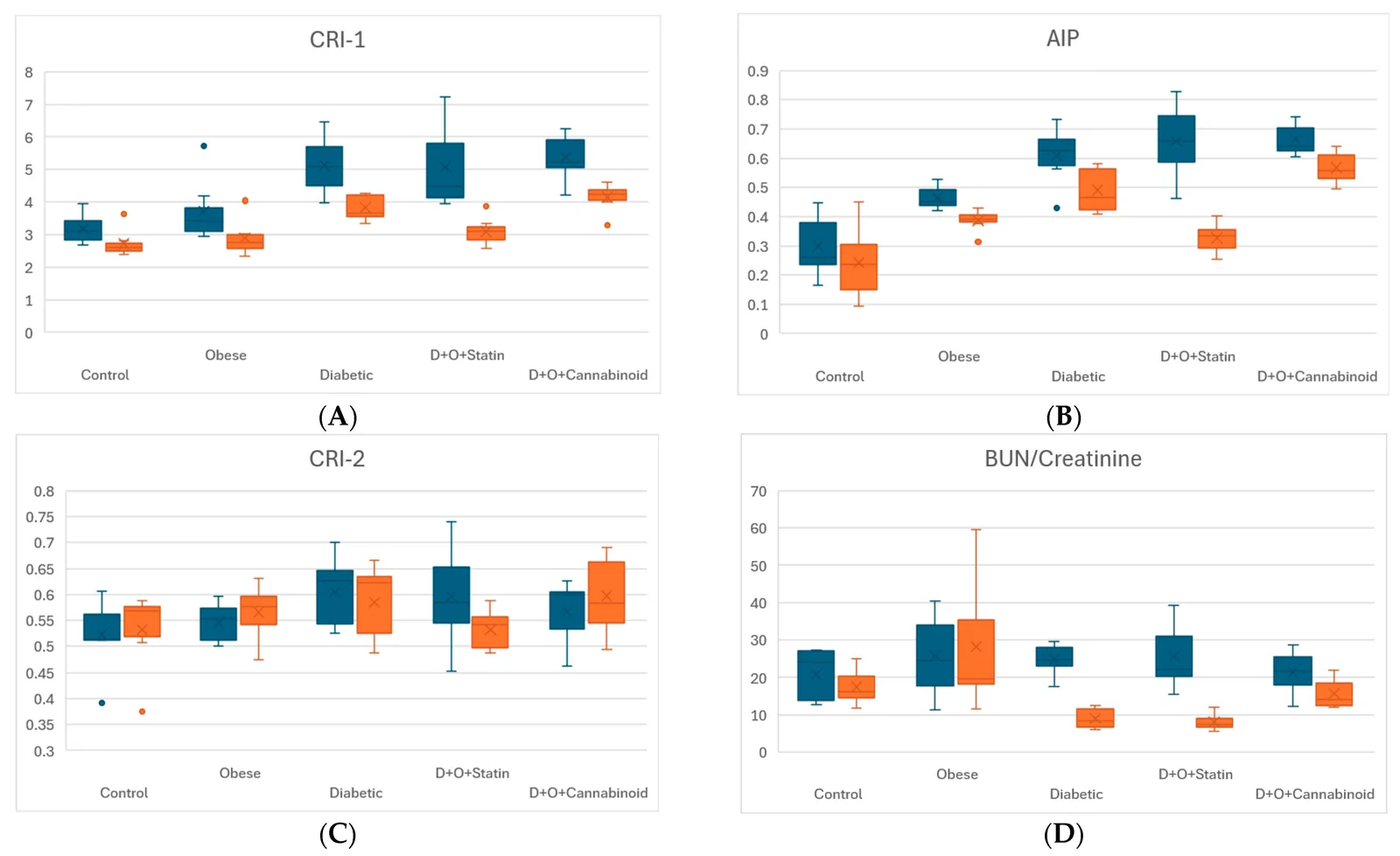
(**A**–**D**). Before–After boxplots: blue, initial values; orange, final values; paired boxplots for each of the five groups, identified below each pair. Left upper corner, (**A**) Castelli Risk Index 1. Right upper corner, (**B**) atherogenic index of plasma. Left lower corner, (**C**) Castelli Risk Index 2. Right lower corner, (**D**) blood urea nitrogen/creatinine ratio.

The visual depiction of the initial and final values for the aforementioned indices is found in Figure 4A–D.

### 3.5. Liver Safety Assessment Through Derived Indices

We evaluated the de Ritis ratio and its fibrosis-focused variant, ASAT/sqrt(ALAT). de Ritis ratio showed a statistically significant increase in both G-IV and G-V at the end of the experiment, while in G-III, this index was virtually unchanged. The effect size with both interventions was large, as shown by a Hedges’ *g* = 1.836 with statins, and a Hedges’ *g* = 0.861 with cannabinoids. However, comparison of the effect sizes was statistically significant (*p* = 0.002). This is strengthened by the wide-apart values of mean of differences—with statins, MD: 0.266, with cannabinoids, MD: 0.043.

The fibrosis-focused variant showed an increase in groups G-III to G-V, but only in G-IV did it attain statistical significance. The effect size with statins was large in G-IV, as shown by a Hedges’ *g* = 2.755 with statins, but moderate in G-V, with a Hedges’ *g* = 0.505. Comparison of effect sizes was statistically significant (*p* < 0.001). The mean of differences with statins was MD: 0.400, while with cannabis, it was MD: 0.034.

### 3.6. Obesity Presence Following HFD

**Figure 5:**
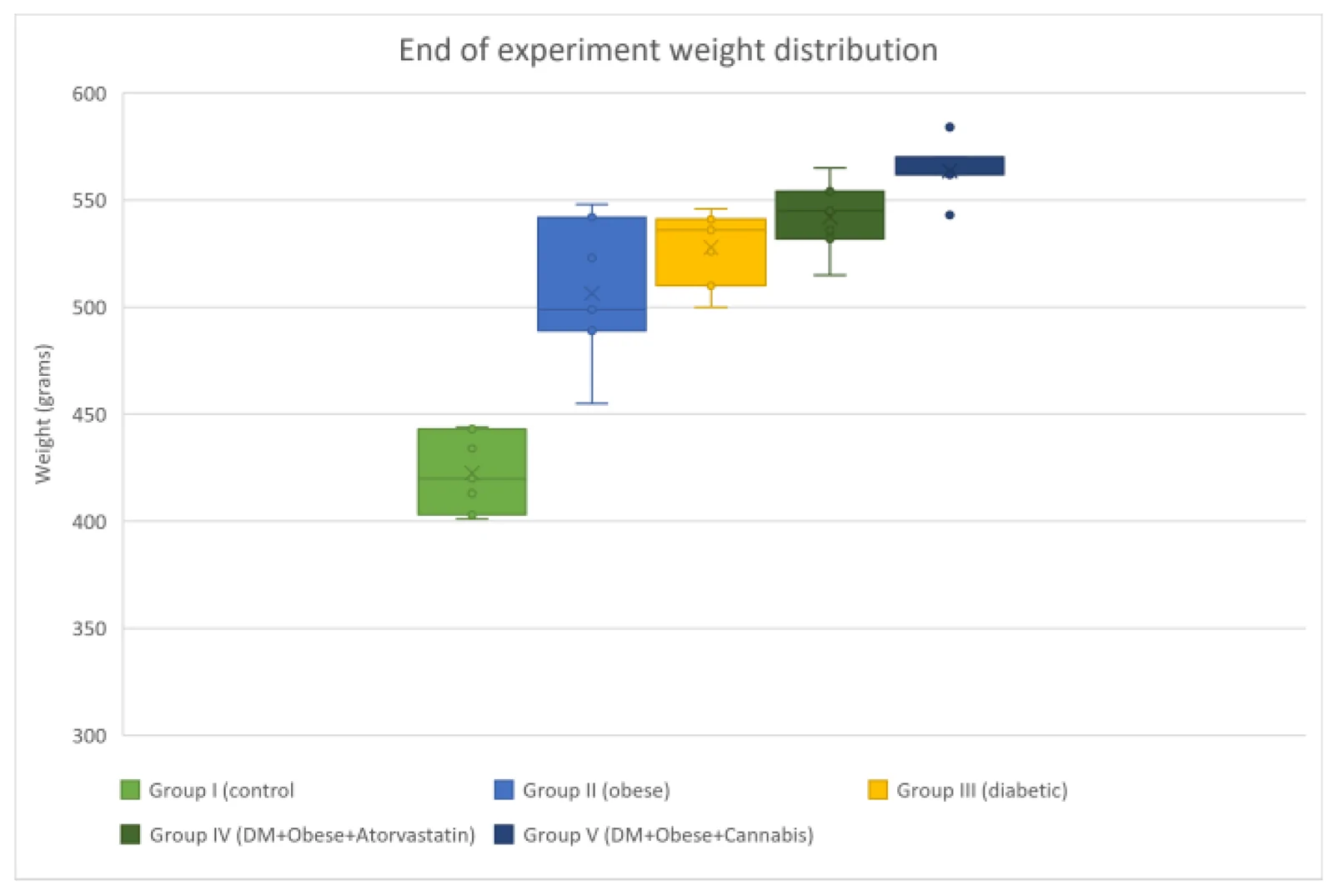
Weight distribution at the end of the experiment, by group.

Obesity has been achieved using High-Fat Diet, 2% rich in cholesterol. The detailed composition of the diet is available elsewhere [29]. The confirmation of obesity inducement is shown in Figure 5 below; weight distribution is shown at the end of the study.

The control group sits at the lowest mean value, MD: 423; IQR [403; 443] grams, at a statistically significant difference before the next nearest group, G-II, MD: 506; IQR [489; 542] grams. The weight of the control group rodents is in line with the literature’s expectations at the age of 7 months for a male Wistar. G-II, G-III and G-IV sit at similar weights, while G-V sits at a statistically significant difference compared to G-IV, of MD: 564; IQR [562; 570].

## 4. Discussions

### 4.1. Assessment of the Impact of Treatment with Atorvastatin or THC on the Lipid Profile and Atherogenic Indices of Plasma

This study examines the longitudinal effects of two interventions in a murine model of obesity and type 2 diabetes: conventional lipid-lowering therapy with atorvastatin (G-IV) and THC-based treatment (G-V). Their impact was assessed through changes in the lipid profile and plasma atherogenic indices—as primary outcomes.

#### 4.1.1. Contrasting with Atorvastatin, Cannabis Extract Did Not Significantly Alter the Basic Lipid Profile

The levels of LDL-cholesterol, total cholesterol, and triglycerides showed no statistically significant change comparing before and after the treatment in G-V treated with cannabis plant extract. With statins, LDL-Col did not exhibit a significant change following treatment, but there was a clinically and statistically significant difference in total cholesterol and triglycerides. Although apparently disconcerting, the significant increase returned by total cholesterol in G-IV likely stems from the major increase in HDL-Col, in the absence of a significant difference in the predominant cholesterol fraction—LDL-Col.

LDL-Col did not show a significant change with either treatment due to the wide spread of effect around the mean; therefore, its change with either treatment can be mostly attributed to chance, or at least two mutually canceling actions stemming from therapy, diet and induced metabolic dysfunctions. Total cholesterol was increased solely in the group treated with statins, and no other group came close to this effect, likely attributing the effect to treatment alone. Triglycerides showed a clinically, but not statistically, significant decrease with statin treatment, an effect that was not statistically different from the change exhibited by the untreated obese and diabetic groups G-II and G-III, questioning the true effectiveness of statins in alleviating this parameter.

The lack of effect on LDL-Col level, an occurrence commonly regarded as statin resistance, despite general practice acknowledgement, is most often blamed on lack of adherence to treatment in the general population [38]. The patients who fail to reach LDL-C target values despite the best available therapy are considered to be statin-resistant [39]. In our experiment, treatment adherence is not an explanation; therefore, resistance to statins may stem from differences in drug absorption, transport, intrahepatic drug metabolism, or within other organs, and drug excretion mechanisms. Furthermore, the presence of satellite comorbidities such as hypertension and overt inflammatory pathway activation—IL-1β is mainly responsible—may require higher statin doses for the expected effect to occur, at least in humans [40]. Clusters of metabolites involved in pathways not directly connected with cholesterol metabolism likely play a role in modulating the response to statin therapy, as demonstrated by comparison of simvastatin responders’ metabolic profile: correlation with LDL-Col values was achieved for cystine, urea cycle intermediates, ornithine, citrulline and lysine [41]. These former amino acids share plasma membrane transporters with arginine, the rate-limiting substrate for nitric oxide synthase, key effector of cardiovascular protection.

Murine models of obesity and diabetes typically show elevated serum triglycerides and increased atherogenic plasma fractions [42]. However, rats differ from humans in several aspects of lipid metabolism, which may attenuate the severity of the atherogenic phenotype. Their enhanced fatty acid metabolism and increased clearance of triglyceride-rich particles confer a degree of intrinsic resistance to the development of dyslipidemia compared with human subjects [43,44]. This species-specific lipid handling may explain why, in our experiment, atorvastatin did not return a relevant reduction in LDL-Col. In rats, regulatory differences in LDL receptor activity—likely arising from epigenetic regulation, together with compensatory changes in endogenous cholesterol synthesis, which takes the use of the trans-intestinal pathway for cholesterol clearance—may partially offset the expected lipid-lowering effect of atorvastatin [45,46,47,48]. By contrast, THC administration did not induce notable changes in total cholesterol or triglyceride concentrations, suggesting that its metabolic effects may be mediated primarily through modulation of lipoprotein pathways rather than through direct inhibition of de novo fatty acid synthesis [49,50,51].

Cannabis extract treatment appeared to not exert any significant effect on triglycerides. This observation may be the result of the conditions of our experiment. First of all, the duration of the experiment was limited to 4 weeks (or 30 days), which is somewhat lower than the administration window of THC in both animal and human studies [24,52]. Secondly, the ratio of THC to CBD in our experiment favored the first compound. Other studies evaluated a more balanced treatment, noting a favorable effect on triglyceride concentration [53]. Nevertheless, adipocyte storing and increased lipolysis activity are exacerbated even under a short duration treatment consisting of solely THC. This may partly explain the noted lack of effect on triglyceride concentrations, in spite of the anti-inflammatory effect of cannabis plant extract [50].

#### 4.1.2. Is the Effect upon HDL-Col as Impressive as It Seems?

In the present study, HDL-Col increased in both treatment groups, with the most pronounced and statistically significant rise observed in the atorvastatin-treated group. The control groups G-I to G-III also exhibited a paradoxical improvement in HDL-Col, but the magnitude of effect was significantly higher in G-IV. An even higher magnitude of effect was noted in G-IV compared to G-V. HDL-Col showed clear improvement with statins, an effect size which far surpassed the change in the untreated groups. HDL-Col showed an improvement with cannabis plant treatment, but the change was marginally higher than in the untreated diabetic group, lacking statistical significance, and questioning the impact of the cannabis plant versus populational dispersion.

HDL-Col has a well-established cardioprotective role, primarily through reverse cholesterol transport, whereby cholesterol is mobilized from peripheral tissues and macrophages and directed to the liver for biliary excretion [54]. These findings are consistent with previous reports describing favorable effects of statins on HDL in obese rodents, mediated by microbiome modulation [55]. In the cannabis-treated group, the increase in HDL-Col may be explained by THCA-A-mediated modulation of CB1R and activation of CB2R. Activation of the former modulates the effects of CB1R stimulation in certain tissues (i.e., central nervous system). Epigenetic regulator for several shear stress-related molecules (PPAR-γ, TRP channels), CB2R may be responsible for the observed attenuation of low-grade systemic inflammation seen in metabolically challenged models [56,57,58,59]. Furthermore, CBGA has been reported to act as a dual peroxisome proliferator-activated receptor (PPAR) α/γ agonist [60]. PPAR-α is a key regulator of lipid metabolism, while PPAR-γ is involved in glucose homeostasis and adipocyte differentiation, and dual agonists are studied as potential candidates for metabolic syndrome and type 2 diabetes. CB1R amplifies and is expressed in relation to shear stress but may be more directly involved in determining HDL-Col levels [18,61]. Interestingly, cholesterol itself increases the basal activity levels of CB2R specifically, casting doubt regarding the desirability of drastic cholesterol reduction for this mechanism [62].

#### 4.1.3. The Effect upon Atherogenic Indices Is More Heterogeneous

Plasma atherogenic indices provide a more integrated assessment of cardiovascular risk than isolated lipid parameters, capturing the balance between proatherogenic and protective lipid fractions. In the present study, AIP improved significantly after both interventions, as reflected by lower values at the end of the experiment. Although the effect size was large in both G-IV and G-V, the magnitude of change was significantly greater after atorvastatin treatment than after cannabis plant administration. CRI-1 also decreased significantly across all study groups, including the control groups; however, the reduction was more pronounced in the atorvastatin-treated group than in the cannabis-treated group. By contrast, CRI-2 showed a more heterogeneous pattern, with atorvastatin and THC shifting values in opposite directions. Neither intervention produced a statistically significant within-group change in CRI-2, although the between-treatment comparison remained significant after baseline adjustment, likely stemming from the divergent directions of effect.

AIP is independently associated with extent of coronary artery disease by reflecting the balance between triglyceride-rich lipoproteins and HDL-Col, although possibly missing the contribution of LDL-Col as a highly atherogenic fraction [63]. Regardless of risk, AIP shows moderate prognostic ability for MACE and metabolic syndrome, highlighting the desirability of lowering this index through medication [64,65,66]. Research on human subjects confirms the association of lower AIP values with coronary angiography plaque findings following statin treatment regimen combined with antioxidants [67]. AIP shows significant association with coronary artery permeability—both stenosis extent and perfusion in the absence of hemodynamically relevant stenosis [68,69]. Statins delay plague progression by tilting the balance between HDL-Col and LDL-Col towards higher lipoprotein particle average size. In G-IV, the significant reduction in AIP supports the antiatherogenic effect of atorvastatin in obese and diabetic animals. The concomitant decrease in CRI-1 further suggests a lower susceptibility of the endothelium to atheromatous plaque formation in this treated model. By contrast, the absence of a statistically significant change in CRI-2 indicates that the intervention did not substantially modify the LDL-Col/HDL-Col relationship.

Castelli Risk Index is associated with coronary artery lesion severity in various states of glucose metabolism, extending its potential to prediction of cardiovascular mortality in diabetic patients [70,71]. The predictability potential of CRI is not limited to coronary arteries, as it can be independently used to identify vulnerable carotid plaques in patients at risk for ischemic stroke [72]. Overt endothelial dysfunction is followed by coronary artery lesion or major adverse cardiovascular events; therefore, endothelial status may be predicted using these surrogate lipid profile-derived atherogenic indices. Caution should be taken following a MACE, as none of the above indices could predict early mortality following a non-ST elevation myocardial infarction [73]. Following cannabis administration, reductions in AIP and CRI-1 suggest a partial improvement in endothelial dysfunction associated with obesity and diabetes. These findings indicate that cannabinoid-mediated mechanisms may contribute to systemic vascular protection, although the magnitude remained lower than that observed with conventional lipid-lowering therapy.

Our study shows a decrease in atherogenic index of plasma in all groups at the end of the experiment, an effect determined by the incompletely understood increase in HDL-Col, which is in opposition with the literature concerning obesity models; not only should obesity shift the distribution of lipoproteins towards lower density ones, but also it affects the distribution within the HDL-Col class towards lower dimensions, together with alteration in adipokine levels [74]. Diabetes similarly impacts the levels and anti-inflammatory activity of HDL-Col particles [75]. Certain components in the obesogenic diet may alleviate HDL-Col decrease (e.g., olive oil), but these constituents were not part of the HFD employed in our experiments. We consider the paradoxical increase in HDL-Col in G-II and G-III a limitation of our study. However, comparison of the Before–After differences favors the statin treatment in a statistically significant fashion against the changes noted in the control groups—at least for AIP and CRI-1. Taken together, these results show that cannabis plant treatment improved selected atherogenic risk markers, particularly AIP and CRI-1, and was associated with increased HDL-Col levels in obese and diabetic male Wistar rats. However, the overall antiatherogenic efficacy of cannabis plant remained inferior to that achieved with atorvastatin.

### 4.2. Renal Function Impact of Interventions

Diabetic nephropathy, a serious complication of obesity and diabetes mellitus, represents one of the major challenges in human clinical practice.

#### 4.2.1. Urea and Creatinine Plasma Concentration Dynamics

In the present study, serum urea concentrations were elevated in G-III and in G-IV before atorvastatin administration, followed by significantly lower values after treatment. Urea concentration displayed a large effect size with statin administration, but comparison of the Before–After relative changes between G-III (diabetics, untreated) and G-IV (statin treated) largely cancels the attrition of urea concentration improvement from statin therapy. In the same note, the slight alteration of creatinine plasma values with cannabis plant treatment was not statistically significantly different than the Before–After relative changes exhibited by the control G-I.

The noticed pattern is consistent with the metabolic and renal consequences of dyslipidemia and chronic hyperglycemia, which are associated with increased protein catabolism and reduced glomerular filtration capacity. Under these conditions, cellular metabolism may shift toward greater amino acid utilization, thereby enhancing hepatic gluconeogenesis and increasing urea production. There is evidence concerning the association between increased blood urea nitrogen and the risk of diabetes in cross-sectional analysis, and this relation holds true within diabetic population for risk of insulin use [76,77,78]. In parallel, free fatty acids may accumulate in proximal renal tubular cells and promote ectopic lipid accumulation, glycolysis shift, focal inflammation, oxidative stress, and interstitial fibrosis, all of which can impair the elimination of nitrogenous waste products [79,80,81]. Key molecules such as acyl-Coenzyme A (CoA) synthetase medium-chain family member 3 (ACSM3) demonstrate the self-fueled cycle by impaired fatty acid oxidation, while potential therapies targeting SGLT-2 and PPAR-γ may partially rescue this metabolic pathway. Prolonged hyperglycemia may also promote polyuria and polydipsia, increase fluid turnover and potentially enhance the renal washout of nitrogenous by-products by the end of the study, particularly in groups G-III to G-IV.

Despite the overlap of diabetes and obesity, the presence of atorvastatin treatment in G-IV prevented an expected increase in creatinine, arising from diabetic nephropathy. Central mechanisms include—but are not limited to—macrophage phenotype shift to M2, oxidative stress inhibition, and inhibition of ferroptosis-mediated cell toxicity [82,83]. A distinct pattern was observed in G-V, where THC administration returned a clinically relevant increase in serum creatinine, despite relatively stable urea values. In T2DM, macrophage dysfunction promotes sustained glycolysis-dependent pro-inflammatory activation, increasing cytokines and nitric oxide. This low-grade chronic inflammatory state, regarded as metaflammation, contributes to insulin resistance, persistent metabolic dysfunction and target organ damage, such as diabetic nephropathy [84]. Cannabis plant mediates pro-inflammatory properties mainly through CB2R receptor activation, which may limit parenchymal pro-inflammatory cytokine signaling by activated macrophages [57,85]. Although changes following CB2R activation are still heterogenous with respect to IL-6, IL-1β and TNF-α, the promise is strengthened by the development of CB2R antibodies as treatment for immune-mediated disorders [86]. However, the anti-inflammatory benefits of cannabis plant administration are likely limited in our model. This stems from the higher increase in creatinine average value in G-V compared to the untreated G-III. Two possible explanations are particularly relevant: local CB1R-mediated hemodynamic effects and competitive interference with tubular creatinine transport.

The endocannabinoid system is known to participate in the regulation of renal microcirculation. CB1R is expressed in podocytes and proximal tubule cells [87]. CB1R activity is increased under hyperglycemic conditions, and mixed treatment with CB1R inverse agonists and RAAS inhibitors has a proven benefit [87,88,89]. Prolonged cannabis use has been demonstrated to induce CB1R downregulation at the central level, but it is likely that, in our model, the dosage and frequency of administration exhibited a deleterious effect of CB1R overactivation. This mechanism may contribute to increased serum creatinine concentrations in G-V, with Before–After differences higher than in the untreated G-III.

Concerning creatinine values, it is worth noticing that the mean relative changes following the experiment were closely matched between control groups, G-I and G-V. Strengthening this observation, the absolute change in G-I is similar to G-V, also shown by the lack of statistical significance for the comparison between changes in these two groups. Despite value dispersion being tighter around the mean (smaller SD for relative change per specimen) for G-V, the effect on creatinine cannot be fully attributed to cannabis plant treatment.

#### 4.2.2. Interpretation of the BUN/Creatinine Ratio

There was a statistically significant difference between the two interventions for the absolute effects from a Before–After perspective (*p* = 0.04). Since neither intervention achieved large effect size, the significant difference between them likely stems from the opposite direction of their effect. It is worth pointing out the direction of the parameters included in the formula of this index at the end of the experiment; while in G-IV, urea dropped significantly and creatinine remained virtually constant at the end of the experiment, in G-V, urea values decreased—but to a lower extent—while creatinine values increased—although not attaining statistical significance.

The BUN/creatinine ratio has been traditionally used to differentiate renal failure etiologies, especially in acute or acute-on-chronic settings. The forementioned ratio exhibits a U-shaped relation with mortality and is independently associated with incident T2DM in wide populations [90,91]. In the cannabis-treated group, the observed decrease in this ratio argues suggests that cannabis may influence renal handling of creatinine and water reabsorption. CB1R is likely involved, as its blockade restores serum creatinine concentrations in several animal models. Higher cannabis doses target peripheral CB1R-mediated vasopressin inhibition and reduced water reabsorbtion, while lower cannabis doses reduce urine output through central CB1R modulation [92]. Overall, these findings indicate that classical markers of nitrogen retention should be interpreted cautiously in the context of cannabis exposure, as their values may be shaped not only by glomerular filtration, but also by cannabinoid-related effects on tubular function and renal clearance.

### 4.3. Safety Assessment of Liver Function, Fibrosis Indices, and Glycemic Profile

Analysis of liver enzyme profiles and glycemic homeostasis aids in interpreting the pleiotropic effects of lipid-lowering therapy. In the present study, atorvastatin treatment in obese and diabetic rats revealed a pharmacological paradox: improvement in the glycemic profile occurred alongside worsening liver function and fibrosis-related indices.

#### 4.3.1. Liver Enzyme Levels

G-IV returned a large effect size upon both ALAT and ASAT following the treatment, far outweighing the populational dispersion revealed by the untreated groups. Group V, however, showed modest changes, not significantly different from the changes exhibited by any of the three untreated groups, voiding any possible deleterious effect of cannabis plant extract in this model on the basis of these enzymes.

Atorvastatin administration produced a significant increase in liver enzyme levels in G-IV, suggesting hepatocellular injury in the setting of non-alcoholic steatohepatitis associated with obesity and T2DM. Mild, asymptomatic elevations in ALAT and ASAT following statin therapy are generally expected, and this effect may be amplified when hepatic steatosis is already present. As a lipophilic molecule, atorvastatin can passively diffuse into hepatocytes, where it inhibits HMG-CoA reductase and reduces the synthesis of both cholesterol and coenzyme Q10, a key component of the mitochondrial respiratory chain [93]. This disruption may promote mitochondrial dysfunction, increased reactive oxygen species generation, and oxidative stress. Together, these processes can destabilize hepatocyte membranes, contribute to focal necrosis, and facilitate the release of liver enzymes into the systemic circulation. While treatment interruption is recommended in those patients with enzymes over three times the upper limit of normal range, most instances of increased ASAT or ALAT concentrations resolve spontaneously and are not commonly associated with altered liver histology [94].

In the case of cannabis-treated rats, the changes in ASAT and ALAT were not clinically or statistically significant, possibly denoting a preventable liver damage through cannabis administration. This observation is in line with recent research advancing the potential benefit of whole cannabis plant towards reducing the levels of pro-inflammatory cytokines and tilting the balance of polyunsaturated fatty acids towards omega-3 predominance [95,96]. The dosage and balance between the active compounds within the cannabis plant fraction are crucial, as some compounds (i.e., THC) are metabolically neutral in comparison to others (CBD) [52,97].

#### 4.3.2. De Ritis Ratio and the More Fibrosis-Focused Derived Index

The significant increase in the de Ritis ratio observed in G-IV points towards other sites of injury than solely the liver. ALAT is primarily a cytoplasmic enzyme and is generally associated with acute hepatocellular damage, whereas ASAT has both cytoplasmic and mitochondrial localization in most cells as well as hepatocytes. High de Ritis values may indicate either severe hepatocellular injury or extrahepatic damage. Retrospective studies focused on statin-associated liver enzymes generally show higher values for ALAT than ASAT [98,99]. In our study, the higher than 1 de Ritis ratio may be the consequence of muscle breakdown during statin treatment, but this cannot be supported by other biochemical or physical parameters. de Ritis ratio has been independently associated with a worse prognosis in individuals with coronary artery disease—a common finding in diabetic and obese individuals—in patients with elevated liver enzymes [100,101].

Although statins have been reported to exert antifibrotic effects in some experimental and clinical settings, this protective effect may be attenuated or altered in the context of metabolic syndrome, as reflected by the findings in G-IV. The pre-existing accumulation of triglycerides and free fatty acids within hepatocytes may act synergistically with atorvastatin-induced mitochondrial stress, overriding the statin-driven beneficial peroxisomal and mitochondrial oxidation capacity [94]. These findings suggest that, in obesity associated with diabetes, the cardiovascular benefits of atorvastatin may be undercut by a substantial hepatic burden, namely, activation of fibrosis-related pathways.

In the present study, the fibrosis-focused index derived from FIB-4 (i.e., missing platelet count from the formula) significantly increased in G-IV, in comparison to virtually no change in G-V treated with cannabis plant. Our findings apparently contradict studies on numerous human cohorts which consistently report a protective effect of statins on liver histology, a proportional effect to statin usage duration and dosage [102,103]. This may arise from extensive baseline metabolic dysfunction-associated steatotic liver disease, a baseline missing in our study due to the poor survival of the untreated pilot study group of diabetic and obese rats.

Cannabis plant appeared to not return a significant effect upon ASAT/ALAT ratios, but there was a slight increase in de Ritis and FIB-4 derived indices. The literature takes a cautious approach regarding the safety of cannabis-derived compounds upon liver status, especially CBD, as far as considering the CBD-associated liver enzymes increase a common adverse drug event [104,105]. This does not imply CBD—or cannabis—should be regarded as hepatotoxic compounds.

#### 4.3.3. Evolution of Glycemia After the Experiment

In the present study, atorvastatin treatment was associated with a statistically significant—yet clinically limited—improvement in the glycemic profile of G-IV. It is worth noting the lack of statistical significance for the comparison of Before–After changes between the pairs G-II and G-IV, and G-III and G-IV. Since neither G-II nor G-III received treatment, the reduction in glycemia in G-IV after the experiment is not the result of statin therapy alone, therefore limiting the implication of the noted effect. Statin itself is unlikely to have determined an improvement in glycemia values.

The observed improvement on glycemia is contrary to several human studies which have linked atorvastatin therapy to an increased risk of T2DM. Both short- and long-term statin therapy (i.e., 10 weeks up to 9 years) appear associated with increased risk of T2DM and worse insulin sensitivity, both in trials and prospective cohorts [106,107,108]. The mechanism, independent of statin treatment duration, involves decreased expression of proteins involved in glucose consumption—glucose transporter 4 (GLUT4) and insulin signaling—of insulin receptor subunit β. Short-term statin treatment reduced HNF4 in hepatocytes of model animals, mediating the increased expression of PAQ9. The HNF4α-PAQR9-STUB1-PPM1α axis downregulates the Akt pathway downstream from the insulin receptor, strengthened by the beneficial effect of weight loss and PAQ9 receptor inhibition [109,110,111]. Most importantly, the previously noted effects are dependent on statin type—with pravastatin and rosuvastatin showing no correlation.

The eating pattern of the animals under HFD was different across groups, impacting both their rhythm and their final weight. High-Fat Diet has been proven palatable by male rodents under free water access conditions, and there is human evidence that HFD can help regulate glycemia levels in diabetic individuals [112]. However, animals under cannabis plant extract treatment demonstrated the highest final weight, and they showed a handler presence-dependent food-searching behavior, with the highest activity approximately one hour after treatment administration, while the handler was still inside the room (this was evaluated using continuous camera monitoring and AI activity detection over 24 h).

### 4.4. Strengths and Limitations

This study is among the few to evaluate the effects of THC-based compounds on endothelial status in a metabolically challenged murine model. The selected model closely reflects key features of the human metabolic profile, including obesity-related type 2 diabetes and associated cardiovascular–kidney–metabolic alterations. The design was strengthened by the inclusion of atorvastatin as a standard treatment comparator, enabling a direct assessment of THC-based intervention against an established lipid-lowering therapy. The primary outcomes focused on lipid profile and renal function, both of which are central components of the cardiovascular–kidney–metabolic axis and are substantially disrupted in this rodent model.

However, our study presents several limitations. Fixed compound, dosage and treatment duration may be the most obvious. Furthermore, we did not record behavioral data in the treatment groups, to control for potential adverse effects of THC. Water and food consumption was not recorded sufficiently accurately to allow for meaningful comparison between intervention groups. Body composition analysis was not available, to assess for adipose tissue quantity between interventions. We did not include an obese and diabetic group as a control group for G-IV and G-V because previous pilot studies revealed poor survival of this model. We did not compare it with other compounds from the same class of medication, and we used relatively young animal specimens, which may better tolerate oxidative stress. The small sample size was chosen due to ethical concerns, but the moderate dispersion of values around the mean in most parameters limits the power of our findings. Lastly, the translatability of this model is limited by disease induction design and by the potentially undesired psychiatric effects of cannabis plant administration.

## 5. Conclusions

This study approaches the effects upon lipid profile and atherogenic indices of cannabis plant extract and atorvastatin in a parallel design framework by employing a rat model of obesity and superposed type 2 diabetes mellitus. Atorvastatin impacted most HDL-Col concentration, without a significant change in the rest of the lipid profile components. Noticeably, statin administration improved most—but not all—of the picked surrogate atherogenic indices. However, concerns regarding hepatotoxicity, as reflected by elevated liver enzymes and the de Ritis ratio, underscore potential risks. By contrast, cannabis plant extract administration did not result in lipid profile improvement larger than changes in control groups. The changes in the three atherogenic indices followed a similar profile, with post-treatment differences not significantly different than the larger changes across the control groups.

Future studies should further define THC/cannabidiol pharmacodynamics, with particular attention to renal, hepatic, and neurological effects during longer-term administration at low doses. Studies focusing on dose–response relationships, long-term safety, and behavioral impacts in diverse models will be critical for assessing cannabinoid therapy’s clinical applicability in patients with complex metabolic syndromes, potentially guiding more effective treatment strategies. The neurological potential downsides warrant caution against progressing to human studies.

## Figures and Tables

**Table 1:** Group design and composition following model induction and allocation.

Group No.	Design and Treatment Administered
G-I	Control, healthy; no treatment
G-II	Obese (High-Fat Diet); no treatment
G-III	Diabetic (Streptozotocin single injection); no treatment
G-IV	Obese and diabetic (High-Fat Diet and Streptozotocin inj.); atorvastatin
G-V	Obese and diabetic (High-Fat Diet and Streptozotocin inj.); cannabinoids

## Data Availability

The original contributions presented in this study are included in the article/Supplementary Materials. Further inquiries can be directed to the corresponding authors.

## Supplementary Materials

The following supporting information can be downloaded at: https://www.mdpi.com/article/10.3390/life16081383/s1, Table S1, raw data used for providing the results. Table S2, *p* values for Before–After absolute value comparison.

## Abbreviations

The following abbreviations are used in this manuscript:

ACSM3	Acyl-CoA Synthetase Medium-Chain Family Member 3
AIP	Atherogenic index of plasma
ALAT	Alanine aminotransferase
ASAT	Aspartate aminotransferase
BUN	Blood urea nitrogen
CBD	Cannabidiol
CBxR	Cannabinoid receptor x
CRI-x	Castelli Risk Index x
FIB-4	Fibrosis index 4
GLUT4	Glucose transporter 4
G-x	Group no. x
HDL-Col	High-density lipoprotein-bound cholesterol
HFD	High-Fat Diet
HMG-CoA	3-hydroxy-3-methylglutaryl coenzyme A
IQR	Interquartile range
LDL-Col	Low-density lipoprotein-bound cholesterol
MACE	Major adverse cardiac event
MD	Mean of differences
PAQ9	Progesterone and adiponectin receptor 9
PPAR-x	Peroxisome proliferator-activated receptors x
PPM1α	Protein phosphatase, Mg^2+^/Mn^2+^ dependent 1
RAAS	Renin–Angiotensin–Aldosterone system
sCr	Serum creatinine concentration
SD	Standard deviation
SGLT-2	Symporter glucose transporter 2
STUB	STIP1 homology and U-box containing protein 1
T2DM	Type 2 diabetes mellitus
THC	Δ9-tetrahydrocannabinol
THCA-A	Tetrahydrocannabinolic acid
TRP	Transient receptor potential channel
